# Implementing services for Early Infant Diagnosis (EID) of HIV: a comparative descriptive analysis of national programs in four countries

**DOI:** 10.1186/1471-2458-11-553

**Published:** 2011-07-13

**Authors:** Anirban Chatterjee, Sangeeta Tripathi, Robert Gass, Ndapewa Hamunime, Sok Panha, Charles Kiyaga, Abdoulaye Wade, Matthew Barnhart, Chewe Luo, Rene Ekpini

**Affiliations:** 1 Department of Health and Nutrition, UNICEF Ghana, Accra, Ghana; 2 Health Section, UNICEF, 3 United Nations Plaza, New York, 10017, USA; 3 HIV/AIDS Section, UNICEF Thailand, Bangkok, Thailand; 4 Case Management Unit, Ministry of Health and Social Services, Windhoek, Republic of Namibia; 5 VCCT and Laboratory Support Unit, National Center for HIV/AIDS, Dermatology, and STD, Phnom Penh, Kingdom of Cambodia; 6 AIDS Control Program, Ministry of Health, Kampala, Republic of Uganda; 7 Division of HIV/AIDS, Ministry of Public Health, Dakar, Republic of Senegal; 8 HIV Section, UNICEF, 3 United Nations Plaza, New York, 10017, USA

## Abstract

**Background:**

There is a significant increase in survival for HIV-infected children who have early access to diagnosis and treatment. The goal of this multi-country review was to examine when and where HIV-exposed infants and children are being diagnosed, and whether the EID service is being maximally utilized to improve health outcomes for HIV-exposed children.

**Methods:**

In four countries across Africa and Asia existing documents and data were reviewed and key informant interviews were conducted. EID testing data was gathered from the central testing laboratories and was then complemented by health facility level data extraction which took place using a standardized and validated questionnaire

**Results:**

In the four countries reviewed from 2006 to 2009 EID sample volumes rose dramatically to an average of >100 samples per quarter in Cambodia and Senegal, >7,000 samples per quarter in Uganda, and >2,000 samples per quarter in Namibia. Geographic coverage of sites also rapidly expanded to 525 sites in Uganda, 205 in Namibia, 48 in Senegal, and 26 in Cambodia in 2009. However, only a small proportion of testing was done at lower-level health facilities: in Uganda Health Center IIs and IIIs comprised 47% of the EID collection sites, but only 11% of the total tests, and in Namibia 15% of EID sites collected >93% of all samples. In all countries except for Namibia, more than 50% of the EID testing was done after 2 months of age. Few sites had robust referral mechanisms between EID and ART. In a sub-sample of children, we noted significant attrition of infants along the continuum of care post testing. Only 22% (Senegal), 37% (Uganda), and 38% (Cambodia) of infants testing positive by PCR were subsequently initiated onto treatment. In Namibia, which had almost universal EID coverage, more than 70% of PCR-positive infants initiated ART in 2008.

**Conclusions:**

While EID testing has expanded dramatically, a large proportion of PCR- positive infants are initiated on treatment. As EID services continue to scale-up, more programmatic attention and support is needed to retain HIV-exposed infants in care and ensure that those testing positive initiate treatment in a timely manner. Namibia's experience demonstrates that it is feasible for a rural, low-income country to achieve high national coverage of infant testing and treatment.

## Background

HIV infection is having an increasing impact on the health of children, threatening to undermine hard-won gains in child survival in countries with high HIV prevalence. Based upon the most recent global estimates, 2.3 million children younger than 15 years of age are living with HIV [1], the vast majority of whom acquired HIV from vertical transmission. Mortality is high among HIV-infected infants in the first months of life and without access to life-saving drugs, including antiretroviral therapy (ART) and co-trimoxazole prophylaxis (CPT), 30% of HIV positive children die in their first year of life and approximately half do not survive until their second birthday [2]. Importantly, there is a remarkable increase in survival if HIV-infected children have access to early diagnosis and treatment [3]. However access is available to a very limited number of children in need with only 15% of exposed infants in low and middle income countries receiving a virologic test and 28% of ART-eligible infants and children receiving ART [4].

One contributing factor to low coverage of ART in infants is that diagnosing HIV in infants requires virologic testing, rather than simpler antibody-based rapid tests that are used in adults. Early infant diagnosis (EID) is done through polymerase chain reaction (PCR) testing of dried blood spots that are collected at peripheral sites and sent to central laboratories. WHO recommends that all infants born from mothers who tested positive during pregnancy should have a blood sample collected for EID testing at four to six weeks of age [5]. This window has been selected because EID testing has >95% HIV sensitivity at this point, which also coincides with the period when most national guidelines recommend the first set of immunizations for infants. In addition, infants <18 months old who present with signs and symptoms of HIV or infants presenting with unknown exposure status should be screened for exposure and if exposed, be rapidly given an HIV diagnostic test.

Though critically important for the survival of HIV positive infants and the Universal Access objectives of national programs, EID requires capacity, participation, coordination, and management of multiple health structures as well as significant logistical, financial and human investment. Due to increased investments by the global community and national governments the number of laboratories providing EID services to the national program increased dramatically in sub-Saharan Africa from 2005 to 2009. However there remain concerns regarding whether existing national programs are effective in identifying HIV-exposed and HIV-infected infants and children and linking them in a timely manner to required care and treatment. This comparative descriptive analysis across four countries in Africa and Asia reviews the early implementation of EID programs as they are in the process of scaling up, with a specific focus on examining when and where HIV-exposed infants and children are being diagnosed and initiated onto treatment.

## Methods

Four countries- Cambodia, Namibia, Senegal and Uganda - were selected to provide a broadly representative sample of HIV care in high and low HIV prevalence countries in Asia and sub-Saharan Africa. All four ministries of health were solicited and agreed to the review both for national program development and to contribute to the global evidence base. In each country under ministry leadership, a desk review of existing documents and data and key informant interviews with key program managers, laboratory staff, and implementing partners were undertaken. Following this, EID testing data was gathered from the central testing laboratories and then complemented by health facility level data extraction which took place using a standardized and validated questionnaire. The focus of the paper is on the EID laboratory testing data and health facility level data that was extracted, but information on how the desk reviews and key informant interviews were conducted is also included below as reference for others who may want to conduct a similar review.

### Desk Review

The desk review included only available nationally endorsed or commissioned documents, plans, and analyses of the data that were focused on EID, PMTCT, and paediatric care and treatment. Documents where EID, PMTCT, or paediatric HIV care and treatment were not a primary focus or which were not endorsed or commissioned by the respective Ministry of Health were excluded. The rationale for these criteria was that the objective was to conduct a focused review of national EID programs, rather than the activities of specific partners or broader child health services. Documents reviewed included national policy documents, national technical guidelines, relevant memos or circulars, national forms and data collection tools used for EID, national testing and procurement data, any existing documentation of the EID system, national scale up plans, and any analyses done to date of EID service. The documents were provided by the Ministry of Health in each country after a request from the UNICEF country offices. This information was reviewed in order to establish the history, policies and current status of the EID program at a macro level. The desk review began prior to the in-country visit and was supplemented by data made available in the first two days in country.

### Key informant interviews

Key informants were defined as persons actively involved in managing, supporting, or providing early infant diagnosis services. This included program managers in the ministries of health at national and district level, technicians in the reference laboratory, health care workers involved in EID and infant care at site level, and partners involved in EID at an implementation level. Feedback gathered included: (1) policy considerations for EID (testing policy, paediatric ART guidelines, user fees, platform selection, and program aspirations for EID); (2) management of EID (planning, capacity, financing supply chain, EID site expansion, logistics management, and M&E); and (3) EID program implementation (infant identification approaches, logistics {specimen transport and communication of results}, site level coordination, and operational challenges.)

### EID laboratory testing data and health facility level data extraction

The fact that early infant diagnosis is a reference test allows for a significant amount of data to be gathered centrally. The EID database at the central laboratory was thus the lynchpin of the review and was used to prepare analyses of testing volumes, age at testing over time, and turn-around time analyses. The available data was extracted from the central database which incorporated data from all laboratories performing EID in each of the four countries. PMTCT/MIS data was gathered at national and regional level.

**Figure 1:**
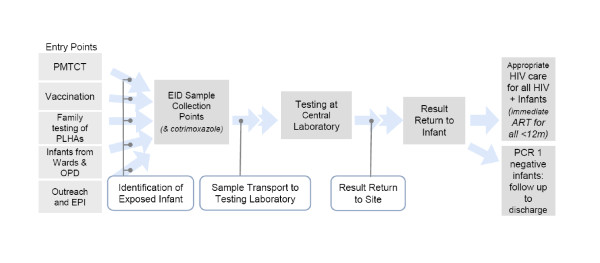
**Infant Diagnosis Service Delivery Continuum**. Note: At the time of the reviews, WHO guidance recommended that all children under 12 months testing positive by PCR be initiated onto ART. 2010 WHO guidance now recommends that all children under 24 months of age that test PCR-positive should be initiated onto treatment.

For the health facility level data extraction, 18 to 25 EID collection sites per country were selected based on health centre type, geography, partner participation and maturity of the EID service. EID collection sites reviewed contributed to variable proportions of total EID tests completed by the national program in the public sector ranging from 36% to 91% (Table 1). The questionnaire, which is attached to this paper as Additional File 1, covered the EID continuum from early identification of the HIV exposed infant (and missed opportunities for identification) through to discharge of the HIV negative baby with a confirmed status or initiation on ART and allowed for the examination of service availability and provision through the EID continuum (Figure 1). Site level data was gathered from site EID registers at different entry points, care and treatment centres, and pharmacies, and was used to complete analyses of turn-around time, result return, and the impact of the EID service. For purposes of this review, age at EID testing was further stratified into three categories (first EID test at < 2 months, at 2-6 months, and at > 6 months). Data collection was conducted between August and December 2009. Data was entered in Excel and analysed using simple frequency distributions. Since the reviews were done using existing data from national programs, the ministries of health and UNICEF staff did not deem it necessary to seek IRB approval or to consult ethical review committees in making this decision.

**Table 1:** Background characteristics of study populations in 4 countries

	1	2	3	4
1	6.5%	13.1%	0.5%	0.9%
2	4	13	14	10
3	20	25	21	18
4	8	1	1	1
5	2	1	1	1
6	17, 602 (36%)	14,148 (56%)	835 (91%)	335 (37%)

Vertical Axis-Countries: 1 - Uganda; 2 - Namibia; 3 - Cambodia; 4 - Senegal

Horizontal Axis - Characteristics: 1 - Adult HIV prevalence - 2009 estimate [7]; 2 - No. of regions in each country covered by review; 3 - No. of health facilities reviewed; 4 - No. of EID testing laboratories testing samples for the national program 5 - No. of EID testing laboratories reviewed 6 - No. of samples (proportion of total cumulative sample volume in public sector) at reviewed sites.

## Results

### EID sample volumes, geographic coverage, and utilization

**Figure 2:**
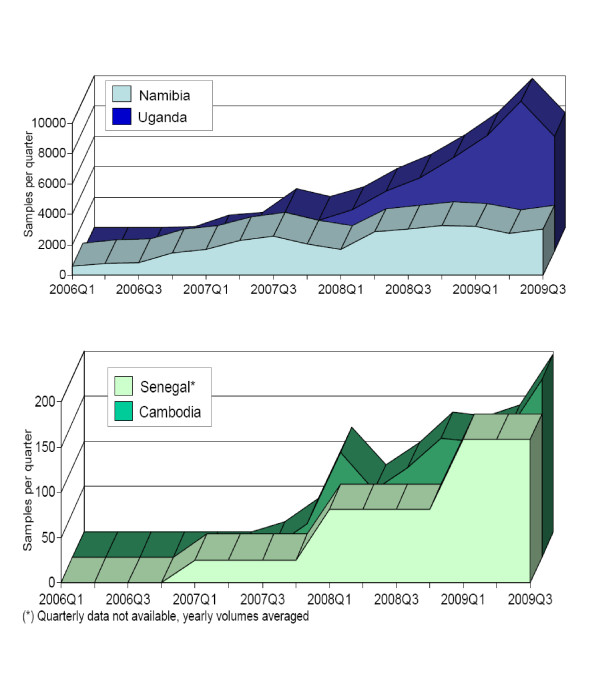
**Samples tested for EID over time for the national programs**.

In the four countries reviewed from 2006 to 2009 EID sample volumes rose dramatically to >100 samples per quarter in Cambodia and Senegal, >7,000 samples per quarter in Uganda, and >2,000 samples per quarter in Namibia. Figure 2 shows that quarterly volumes rose steadily in Uganda, Senegal and Cambodia throughout 2008 and 2009. In Namibia, which had achieved almost universal coverage of EID by 2008, volumes remained steady from 2008 to 2009.

In 2009 in Uganda EID samples were sent to laboratories from over 525 sites across all 4 regions, including all of the Regional Referral Hospitals, 143 of the 161 Health Centre (HC) IVs, 207 of the 955 HC IIIs, and 47 HC IIs. EID services were present at more than half of the ~900 PMTCT sites nationwide and at many more clinics than where paediatric ART was provided (234 sites). In Namibia, EID was available at 205 sites across all 13 provinces, including the large majority of the 35 ART sites and more than 200 PMTCT sites. In Cambodia, EID services were available at 26 sites across 16 of 23 provinces as well as in Phnom Penh and were available at the large majority of the 31 OI/ART Sites and slightly less than half of the 69 Referral Hospitals. In Senegal, EID services were offered at 48 sites across 12 of the 14 regions.

**Figure 3:**
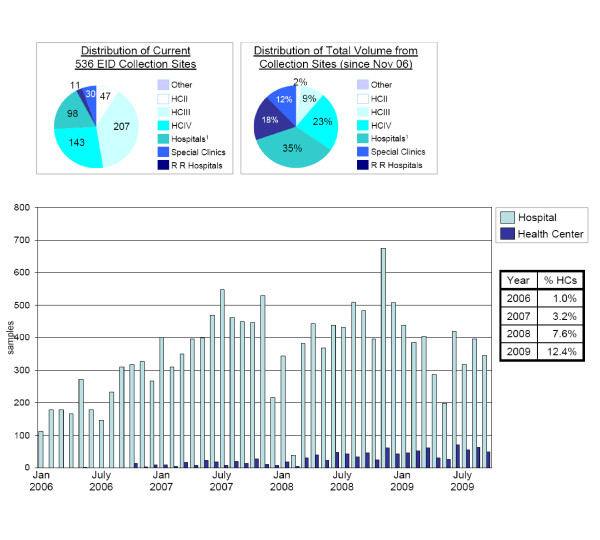
**Utilization of EID by level of health care delivery system**. Top: Availability and uptake of EID by level of health care delivery system in Uganda. Bottom: Number of samples from Hospitals vs. Health Centers in Namibia from 2006-2009.

While expanded geographical coverage across the four countries reviewed brought services closer to the patient, it did not always translate into significantly greater sample collection at lower-level facilities or more even geographical distribution of sample collection. In Uganda lower-level clinics (HC IIs and IIIs) comprised 47% of the EID collection sites nationally, but only 11% of the total testing volume (Figure 3a). Data from Namibia showed ~15% of the national EID sites collected >93% of the total sample volume since the start of the service. (Figure 3b)

### Age at EID testing

**Figure 4:**
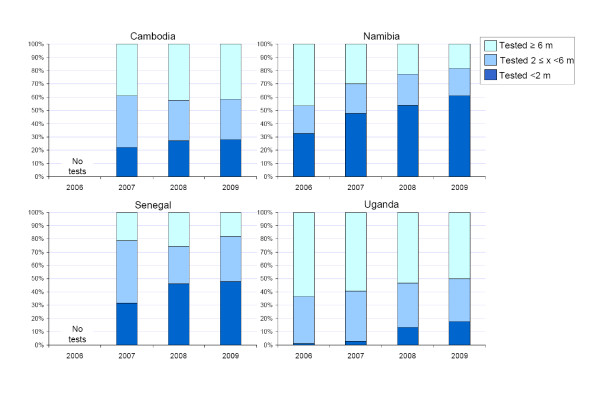
**Time of EID test stratified by age categories (2006-2009)**.

The policies of all countries recommend testing beginning at six weeks or as soon as possible thereafter. In all countries the age of testing was recorded on the testing form, but only in Namibia, did the laboratory coding systematically record the reason for the testing to be either "from PMTCT" or "symptomatic", thus enabling an accurate determination to be made regarding what proportion of testing of infants known to be exposed perinatally was occurring in the context of PMTCT in the first two months of life. In this case of 11,720 total infants were coded as referred from PMTCT services and 1,314 because of having symptoms suggestive of HIV. The median age of testing among infants referred from PMTCT services was approximately 2 months over the life of the program, with a large proportion of these infants being tested well after two months of age. Although the specific reason behind referral for testing in the other three countries was not known, among all infants receiving EID, the proportion of tests that were done in their first two months was less than 50% in 2009 in each country, although this proportion had increased over time in all countries from 2007 to 2009 (Figure 4). The lowest ages at testing across all four countries were seen at the large tertiary paediatric focused centres known to have robust PMTCT and paediatric services and with effective patient follow up systems in place.

### Sample turn-around time

Sample turn-around time (TAT) was analysed from collection at site to laboratory and averaged 1.38 days in Namibia, 5.25 days in Cambodia, and 12.6 days in Uganda over the life of the program, with wide variation between sites. (In Senegal data was not available because date of arrival at the laboratory was not documented in EID database.) Namibia, with the shortest turnaround time, used only one EID testing laboratory, but invested in overnight transportation of all samples from 37 local collection laboratories. Uganda with the most testing laboratories actually had the longest sample transport time. TAT for processing within laboratories averaged 9 days in Namibia, 18 days in Cambodia, and 3.33 weeks in Uganda over the life of the program. In all countries health facility registers did not systematically document the date that the result arrived back at sites and therefore the total TAT from sample collection to result arrival at site could not be measured.

### Models of service organization

There were two main models of service organization observed at facility level for exposed infant care and EID testing. The first is a centralized collection model where services such as MCH, OPD and ART identify exposed infants and refer infants to the site laboratory for EID sample collection. EID sample collection takes place in the site laboratory and the parents of the infants are either told to return to the service from where the infant was referred, or to the laboratory itself for test results. The second collection model that a number of higher volume countries or higher volume sites are implementing is the decentralization of EID sample collection beyond the site laboratory to multiple points within the health care facility such as the ANC, ART centre, OPD, and ward(s). In this model, samples are collected by nurses or doctors within each service. Infants have their EID samples collected, receive exposed infant care, and receive their results all at the same location

### Referral to care and treatment

**Figure 5:**
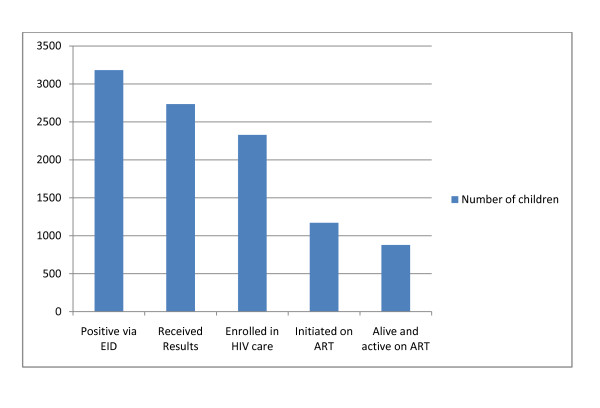
**Outcomes for HIV positive Infants at Reviewed Sites in Uganda**.

Across the sites reviewed, few had robust referral mechanisms in place between testing and HIV care and treatment. In a sub-sample of children in Uganda, Senegal, and Cambodia for whom this data was available we noted significant attrition of infants along the continuum of care post testing. The most complete data and largest sample size was from Uganda (Figure 5). Overall, only 22% (Senegal), 37% (Uganda), and 38% (Cambodia) of infants that tested positive were ultimately initiated on treatment. Among those who did receive their test results and enrolled in HIV care but never initiated ART, some were not initiated because they were: 1) older than twelve months of age at enrolment and not clinically or immunologically eligible to initiate ART; or 2) enrolled at <12 m prior to the guideline changes calling for immediate treatment initiation for infants <12 months testing HIV positive. It is important to note, however, that after WHO issued new recommendations in 2008, Uganda issued a policy change for all infants to be initiated on ART in June 2008, and Senegal and Cambodia similarly changed their guidelines in 2009. Namibia had adapted it guidelines in 2007 to call for immediate ART for all infants even in advance of the 2008 WHO recommendations.

**Figure 6:**
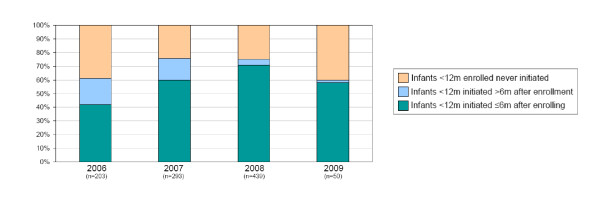
**Initiation of Infants Testing PCR-positive on ART in Namibia**. Data are from the 25 review sites in Namibia and represent the proportion of known PCR-positive infants who were enrolled in HIV care services that initiated antiretroviral therapy <6 m after enrolling in care, >6 m after enrolling, or not at all.

In Namibia, we examined the extent to which PCR-positive infants who were enrolled in HIV care subsequently initiated ART and associated time delays. The proportion of HIV-positive infants initiating ART increased after the new guidelines and delays in time to initiation correspondingly decreased (Figure 6), with more than 70% of known PCR-positive infants initiating ART in 2008 within 6 months of when they were enrolled in HIV care.

## Discussion

We report a dramatic increase in EID testing volumes between 2006 and 2009 in four countries which represent a mix of high (Namibia, Uganda) and lower (Cambodia, Senegal) HIV prevalence and diverse health system capacities. However, while the number of tests expanded rapidly, the public health impact was far from optimal. In 3 of the 4 countries only 22% to 38% of infants testing positive by PCR initiated ART. It is important to recognize that the data reviewed covered a period of time when international guidelines on when to start treatment in infants were changing and therefore not all infants were eligible for treatment at the time they tested PCR positive. However, even after guidelines had been changed, a substantial proportion of PCR-positive infants were still not being initiated on ART at the review sites. The exception to this was Namibia which has been able to achieve high population-based coverage of both infant testing and treatment. Namibia's success demonstrates that it is feasible for EID services to contribute to a large public health impact in a highly rural, low-income country. These observations underscore that it is critical to focus more attention on ensuring that all infants who test positive rapidly initiate ART.

In order to increase EID testing coverage, many countries have prioritized decentralizing the service both geographically and through various levels of the health system. This review found that decentralization alone is not sufficient to greatly increase utilization of services at lower-level sites. To improve uptake of testing in the first two months of life, infant follow up must be underscored in PMTCT counselling. With high coverage rates in many countries for the first childhood vaccination, HIV programs have been working to strengthen the linkage between EPI (Expanded Programme on Immunizations) to enable earlier EID. However these efforts can only succeed when practical operational guidance on provider initiated counselling is coupled with measures such as coded documentation of mothers' HIV status on health cards to enable the identification of infants needing EID.

Among infants with PCR-positive results, very high rates of loss to follow up occurred in the postnatal period. Many caregivers never received the EID results, and very few sites reviewed had robust mechanisms in place to follow up with infants whose parents or caregivers did not return for results. Standard operating procedures are needed by which sample collection and result return are closely linked to counselling, clinical care, and to follow up. It is critical for all sites to have a clear understanding of the EID sample and result flow, the patient flow, and who is responsible for key infant care tasks.

In this regard, the reviews identified two broad types of approaches to organization of EID services, each of which potentially poses challenges for retaining infants. In Senegal and Cambodia, the site laboratory technicians manage the EID supplies, sample collection, record keeping, dispatch of samples, and receipt of results. Having skilled laboratory technicians can facilitate the transportation of samples and communication with referral laboratories, but not having clinical providers in charge of coordinating the service can result in challenges with regard to post-test counselling, exposed infant follow up, and returning the results to caregivers and service providers. On the other hand, the model utilized in Namibia and Uganda in which EID was offered in multiple clinical units within a site (e.g. ART, MCH, wards) requires significant site organization for consumables and registers. With multiple collection points the logistics of getting all samples to the referral laboratory and all results to their correct collection point is challenging.

In light of these difficulties, a third approach to the organization of EID services may be useful in some contexts, which includes aspects of both the centralized and decentralized collection models. To address some of the problems noted in the review, Uganda implemented an EID strengthening pilot in which a single centralized care location is designated where exposed infants receive comprehensive longitudinal care (including CPT, infant feeding counselling and support, ordering the EID tests, returning the results, and follow-up until enrolment in ART or discharge upon a final confirmed negative test.) Sites pick the location of the EID care point themselves by deciding which unit they believe is best positioned, which may be ART, OPD, or MCH clinics - in most cases sites have chosen ART clinics. HIV-exposed infants presenting at other entry points are all referred to and managed by this one designated care point. A focal person is designated to be responsible for infant follow-up at the EID care point, and monitoring tools, job aids, and mentoring are provided for the care point and referring units. Uganda has subsequently reported strong improvements in the proportions of eligible infants receiving test results and initiating ART at sites that have implemented this approach [6], and based upon this successful pilot is now scaling up this approach nationally. While this has worked well in the Ugandan setting, the optimal approach to organization of services may vary in different settings depending on factors such as health system capacity and HIV prevalence.

Systematic referral mechanisms are also needed to ensure PCR- positive infants are enrolled on ART. However, these were not present at most sites providing EID services. Indeed, despite the co-location of testing and HIV care and treatment in the same hospital (and sometimes the same unit), a high rate of attrition occurred between testing and enrolment in HIV treatment for those infants testing positive by PCR. The little data available indicates that, even for the small number of PCR-positive infants that were initiated on treatment, substantial delays were common between when caregivers received results and when infants were initiated on treatment, with many PCR-positive infants being enrolled in HIV care but never HIV treatment. Though the reasons for delays and loss-to-follow-up between enrolment in HIV care and enrolment on ART cannot be determined from the current analysis, possible contributing factors may be the requirement in some cases for several adherence counselling visits prior to ART initiation, the desire of clinicians to obtain a baseline CD4 prior to initiating ART, or the fact that some sites provide care for PCR-positive infants (mainly CPT and infant feeding support) but have to out-refer to other sites that offer ART. This data highlights the urgency of streamlining current processes for initiating ART in infants to avoid such delays which are resulting in increased mortality in these highly vulnerable children. Where feasible, decentralizing ART to lower-level clinics that also provide MCH and EID may also have potential to improve retention through enabling a "one stop shop" where HIV treatment can be initiated without referral to another site or hospital service.

There are several limitations to the conclusions that can be drawn from this retrospective multi-country review. Firstly, the data was routine programmatic data and mostly cross-sectional. Estimating the proportion of infants who were lost-to-follow-up at different points of needed care in the postnatal period therefore required the linking of data from several different sources of routinely-collected data because sites and national programs do not follow HIV-exposed infants longitudinally. However significant efforts were made to eliminate repeat PCR documentation to ensure no infants are double counted. The possibility exists that some of the infants that were estimated as lost-to-follow-up received services, but that this was not recorded either because care occurred at other sites that were not covered by the review or because of the suboptimal quality of the routinely collected data at the sites that were reviewed. We also did not seek to collect data about care services provided to the HIV-positive mothers, such as whether they were enrolled or retained in HIV care and treatment services. Additionally, given the nature of the review, we could not determine what the reasons were why infants were lost-to-follow-up or if any specific approaches had been used successfully to improve the continuum of care. More in-depth prospective reviews would be helpful for programs to better understand these important issues, and the optimal approaches to reduce loss-to-follow-up and ensure timely ART initiation. Encouragingly, since the reviews the countries involved have continued to make improvements in their programs, including taking action to address the high rates of loss-to-follow-up.

## Conclusion

This review provides useful insights into the functioning of EID services in low resource settings and gives an indication of what changes are needed in order to achieve optimal impact of this intervention. While the volume of EID tests has expanded dramatically in these four countries, a large proportion of HIV-infected infants testing positive by PCR were not subsequently initiated on treatment. In order to improve retention and enable the achievement of high coverage of paediatric treatment it is critical for EID services to be fundamentally linked to PMTCT and MCH as well as to ART programs. Shifting focus from the laboratory aspects of EID alone to the full package of HIV-exposed infant care should enable EID services to have a much greater impact on child survival in the future.

## Competing interests

The authors declare that they have no competing interests.

## Authors' contributions

AC made substantial contributions to design of the review; the analysis and interpretation of data; and the drafting of the manuscript. ST made substantial contributions to design of the review; the acquisition, analysis and interpretation of data; and the drafting of the manuscript.

RG made substantial contributions to design of the review; the analysis and interpretation of data; and revising the manuscript for important intellectual content. NH, SP, CK, and AW made substantial contributions to the design of the review and the acquisition, analysis, and interpretation of data. MB made substantial contribution to the analysis and interpretation of data and the drafting of the manuscript. CL and RE made substantial contributions to the design of the review and the analysis and interpretation of data. All authors read and approved the final manuscript.

## Pre-publication history

The pre-publication history for this paper can be accessed here:

http://www.biomedcentral.com/1471-2458/11/553/prepub

## Supplementary Material

Additional file 1 **EID Data Collector Tool**. This is a copy of the questionnaire used for the site-level data extraction. It covers the EID continuum from early identification of the HIV exposed infant through to discharge of the HIV negative baby with a confirmed status or initiation on ART. Click here for file

## References

[B1] UNAIDS Chapter 2: Epidemic Update 2010 Report on the Global AIDS epidemic 2010 Geneva http://www.unaids.org/en/media/unaids/contentassets/documents/unaidspublication/2010/20101123_globalreport_en.pdf Available: Accessed April 15, 2011

[B2] Newell ML Coovadia H Cortina-Borja M Rollins N Gaillard P Dabis F Mortality of Infected and Uninfected Infants Born to HIV-infected Mothers in Africa: A pooled analysis Lancet 2004 364 1236 1243 10.1016/S0140-6736(04)17140-7 15464184

[B3] Violari A Cotton MF Gibb DM Babiker AG Steyn J Madhi SA Jean-Philippe P McIntyre JA Early antiretroviral therapy and mortality among HIV-infected infants N Engl J Med 2008 359 2233 44 10.1056/NEJMoa0800971 19020325 PMC2950021

[B4] WHO, UNICEF, UNAIDS Chapter 5: Scaling up HIV Services for Women and Children Towards Universal Access: Scaling up Priority HIV/AIDS Interventions in the Health Sector: Progress Report 2010 2010 Geneva http://www.who.int/hiv/pub/2010progressreport/ch5_en.pdf Available: Accessed 4 October2010

[B5] WHO WHO recommendations on the diagnosis of HIV infection in infants and children 2010 Geneva http://whqlibdoc.who.int/publications/2010/9789241599085_eng.pdf Available: Accessed 15 April 2011 23741779

[B6] Kiyaga C McConnell I Narayan V Elyanu P Kekitinawa A Tripathi S Katureebe C Akol Z Uganda's innovative efforts to improve testing, retention, and care of HIV-exposed infants 2010 XVIII International AIDS Conference, Vienna http://www.aids2010.org/WebContent/File/AIDS2010_Abstracts_Vol_2_Friday_23July_web.pdf Abstract FRLBE104. Available: Accessed 15 April 20011

[B7] UNAIDS Annex 1 HIV and AIDS estimates and data 2009 and 2001 2010 Report on the Global AIDS epidemic 2010 Geneva http://www.unaids.org/en/media/unaids/contentassets/documents/unaidspublication/2010/20101123_globalreport_en.pdf Available: Accessed 15 April 2011

