# Supplementary material for: Implementing services for Early Infant Diagnosis (EID) of HIV: a comparative descriptive analysis of national programs in four countries
Source: BMC Public Health. 2011 Jul 13;11:553. doi: 10.1186/1471-2458-11-553 (PMC3161890; doi:10.1186/1471-2458-11-553)
Supplement: Additional file 1 — EID Data Collector Tool. This is a copy of the questionnaire used for the site-level data extraction. It covers the EID continuum from early identification of the HIV exposed infant through to discharge of the HIV negative baby with a confirmed status or initiation on ART. [file 1471-2458-11-553-S1.DOC]

**Early Infant Diagnosis (EID) for HIV Collection Site Questionnaire**

SITE DETAIL

| **Site Name** |  |
| --- | --- |
| **Type and District** |  |
| **Interviewer and Date** |  |
| **Colleagues Interviewed:** | ***Name Role Email*** |

SUMMARY

| Number of HIV positive pregnant women followed in 2008 |  |
| --- | --- |
| Number of HIV positive pregnant women followed in 2009 |  |
| Date started EID collection |  |
| Infants EID tested in 2007 (PCR 1 in 2007) |  |
| Infants EID tested in 2008  (PCR 1 in 2008) |  |
| Infants EID tested in 2009  (PCR 1 in 2009) |  |
| Est. % of infants tested put on CTX prophylaxis | **% at clinic ___________________**  **% at clinic ___________________**  **% at clinic ___________________** |
| Site Testing Points:  Infants tested by point  Positive Infants by point | |  |  |  | | --- | --- | --- | |  |  |  | |  |  |  | |
| Total number of Infants testing HIV **positive** via EID |  |
| # of HIV Pos infants who received their test results |  |
| Infants initiated on ART at <18 months of age |  |
| Infants still alive and on ART today (initiated at <18m, can be older now) |  |

|  | **Actively on ART** | **In Care** | **Total** |
| --- | --- | --- | --- |
| Children (0-14) |  |  | **As of Q3** |
| Adults |  |  | **As of Q3** |
| TOTAL |  |  | **As of Q3** |

***If not providing either pediatric or adult ART, note referral site_______________***

**I. Site Laboratory- Technician or Lab Manager Overseeing EID**

| **A. EID at Site Level** | |
| --- | --- |
| 1. Staff trained | Role of person Where trained When Practical  ________________________________ [__] yes [__] no ________________________________ [__] yes [__] no ________________________________ [__] yes [__] no  ________________________________ [__] yes [__] no |
| 1. Date first EID sample was collected | *example.* *08-Aug-2007* |
| 1. List all operational EID collection points, what infants they test, and who collects *(role)* | *Collection Point/Location Collector Infants from*  *______________ _____ _______*  *______________ _____ _______*  *______________ _____ _______*  *______________ _____ _______* |
| 1. Other referral test information | *Location Transport & Frequency*  *Of testing Result Mechanism of transport*  CD4 __________ _________ _________  VL _________ _________ _________  *___ _________ _________ _________* |
| **B. Sample Collection** | |
| 1. Where and how do you order additional EID supplies? |  |
| 1. Have you had stock-outs of any EID consumables? | If yes, for how long  [__] gloves ____________  [__] filter paper ____________  [__] lancets ____________  [__] packing envelopes ___________  [__] desiccant packs ____________  [__] humidity cards ____________  [__] other ______________________ |
| 1. How many times a month is EID collection offered? | [__] 1 per month [__] Weekly  [__] 2 per month [__] Every day  *If not all hours of all days, please note when offered___________* |
| 1. Are there any job aids visible in the collection area? | [__] Yes, DBS collection poster [__] Yes, other________________  [__] Yes, infant testing algorithm [__] No |
| 1. When should caregivers return for their results? | [__] After 2 weeks [__] After 1 month  [__] After 3 weeks [__] Other (explain) ________ |
| **C. Sample Preparation and Sample Transportation** | |
| 1. Where is the EID register located? |  |
| 1. How do EID samples leave the site? | [__] Postal or courier service [__] We bring by taxi  [__] We bring by site vehicle [__] Other or partners_____________  *If you answered postal or courier service:*  how often do they come*? __________*  do they come systematically*?* [__] yes or [__] no |
| 1. Where do your site samples go immediately after leaving your site? | [__] Lab (note which)___________________________  [__] To DHO  [__] Other (specify) _________________________________ |
| 1. Which laboratory processes your EID samples? |  |
| 1. Who is responsible for insuring samples get transported? | [__] Lab technician [__] Nurse/Doctor  [__] Other _____ [__] No one |
| 1. What, on average, is the frequency of sample transportation? Has it changed over time? | Every _____ days  Other notes: |
| 1. Is there a minimum number of samples you can send at a time? | [__] Yes [__] No  *If yes, how many: ______________*  *If collector answers yes,* ***please correct them*** *saying there is no minimum batch. Samples must be sent weekly regardless of how many* |
| 1. How often are you in contact with JCRC? | [__] Frequently [__] Monthly  [__] Rarely [__] Never  If you answered yes, how*? ____________________________* |
| **D. Arrival of Result back at Site** | |
| 1. Who receives EID test results at this site? | [__] Laboratory Technician [__] Nurse/Doctor  [__] Other ___________________________________ |
| 1. How do you know results are ready | *Note mechanism* |
| 1. How do you get the results? |  |
| 1. Are **results** **(pos, neg, invalid)** recorded in the EID register? | [__] Always [__] Rarely  [__] Sometimes [__] Never |
| 1. Is **date of result arrival** recorded in the register? | [__] Always [__] Rarely  [__] Sometimes [__] Never |
| 1. What happens next with the results? | *Note full explanation here:* |
| 1. Roughly how many samples have you had that were rejected? | [__________] Samples  How many of those infants were retested?  [__________] Retested |
| 1. Have you had lost/missing results since the beginning of EID up until now? | [__] Yes [__] No  *If yes, how many __________________* |
| 1. Have you provided 2^nd^ PCR tests for PCR 1 negative infants? | [__] Always [__] Rarely  [__] Sometimes [__] Never |
| 1. Have you provided 2^nd^ PCR tests for PCR 1 positive infants? | [__] Always [__] Rarely  [__] Sometimes [__] Never |
| 1. Are ART centers informed of positive test results? | [__] Always [__] Rarely  [__] Sometimes [__] Never |
| **F. Overall Program** | |
| 1. Is there a focal person for EID at this site? | [__] Yes [__] No  *If yes, what is his/her position ___________________* |
| 1. Frequency of EID supervisions | [__] times a year  Who does the EID supervisions? ________________ |
| 1. What are your major challenges with EID? | *Note response* |
| 1. What advice would you give for a successful EID scale up? |  |

Total number of samples from outreach: ___________

Total number of positive samples from outreach: ___________

***Please fill out (1) Quarterly Totals in attached form***

***(2) First half of Positive PCR FORM***

Exposed Infant Entry Points

|  | **MCH/ PMTCT** | **Vaccinations** | **Other_________** | **Hospitalizations** | **ART Center** |
| --- | --- | --- | --- | --- | --- |
| Staff trained in sample collection | [__] yes  [__] sensitized only  [__] no | [__] yes  [__] sensitized only  [__] no | [__] yes  [__] sensitized only  [__] no | [__] yes  [__] sensitized only  [__] no | [__] yes  [__] sensitized only  [__] no |
| How do you identify who to test? |  |  |  |  |  |
| Do you feel mothers accept EID testing? | [__] always  [__] sometimes  [__] never | [__] always  [__] sometimes  [__] never | [__] always  [__] sometimes  [__] never | [__] always  [__] sometimes  [__] never | [__] always  [__] sometimes  [__] never |
| Are samples collected here? | [__] always  [__] sometimes  [__] never | [__] always  [__] sometimes  [__] never | [__] always  [__] sometimes  [__] never | [__] always  [__] sometimes  [__] never | [__] always  [__] sometimes  [__] never |
| If not here, where/how referred? | Where:  If referred, are they accompanied? (y/n) | Where:  If referred, are they accompanied? (y/n) | Where:  If referred, are they accompanied? (y/n) | Where:  If referred, are they accompanied? (y/n) | Where:  If referred, are they accompanied? (y/n) |
| Is CTX given **here** for free to exposed infants | [__] always  [__] sometimes prescribed  [__] rarely/never  [__] referred to: ___________ | [__] always  [__] sometimes prescribed  [__] rarely/never  [__] referred to: ___________ | [__] always  [__] sometimes prescribed  [__] rarely/never  [__] referred to: ___________ | [__] always  [__] sometimes prescribed  [__] rarely/never  [__] referred to: ___________ | [__] always  [__] sometimes prescribed  [__] rarely/never  [__] referred to: ___________ |
| CTX/ Septrim supply | [__]steady  [__]some stockouts  [__]many stockouts | [__]steady  [__]some stockouts  [__]many stockouts | [__]steady  [__]some stockouts  [__]many stockouts | [__]steady  [__]some stockouts  [__]many stockouts | [__]steady  [__]some stockouts  [__]many stockouts |
| Follow up Date given for results? | [__] 2wks  [__] 1 month  [__] other _______ _______________ | [__] 2wks  [__] 1 month  [__] other _______ _______________ | [__] 2wks  [__] 1 month  [__] other _______ _______________ | [__] 2wks  [__] 1 month  [__] other _______ _______________ | [__] 2wks  [__] 1 month  [__] other _______ _______________ |
| Are results returned to patients at same location? | [__] yes  [__] no  *If no,where______* | [__] yes  [__] no  *If no,where______* | [__] yes  [__] no  *If no,where______* | [__] yes  [__] no  *If no,where______* | [__] yes  [__] no  *If no,where______* |
| Who gives result to patient? | [__] Doctor  [__] Nurse [__] Counselor  [__] Other | [__] Doctor  [__] Nurse [__] Counselor  [__] Other | [__] Doctor  [__] Nurse [__] Counselor  [__] Other | [__] Doctor  [__] Nurse [__] Counselor  [__] Other | [__] Doctor  [__] Nurse [__] Counselor  [__] Other |
| Is contact information gathered when sample is collected? | [__] always  [__] sometimes [__] rarely/never | [__] always  [__] sometimes [__] rarely/never | [__] always  [__] sometimes [__] rarely/never | [__] always  [__] sometimes [__] rarely/never | [__] always  [__] sometimes [__] rarely/never |
| System to know who misses follow up appts | [__] yes, agenda  [__] yes, other  [__] no | [__] yes, agenda  [__] yes, other  [__] no | [__] yes, agenda  [__] yes, other  [__] no | [__] yes, agenda  [__] yes, other  [__] no | [__] yes, agenda  [__] yes, other  [__] no |
| Mechanism to follow up patients who do not return | [__] no  [__] yes *If yes, describe* | [__] no  [__] yes *If yes, describe* | [__] no  [__] yes *If yes, describe* | [__] no  [__] yes *If yes, describe* | [__] no  [__] yes *If yes, describe* |
| Who receives post testing counseling here? | [__]Pos infants [__]All infants tested  [__] Not here  [__] Other _____ | [__]Pos infants [__]All infants tested  [__] Not here  [__] Other _____ | [__]Pos infants [__]All infants tested  [__] Not here  [__] Other _____ | [__]Pos infants [__]All infants tested  [__] Not here  [__] Other _____ | [__]Pos infants [__]All infants tested  [__] Not here  [__] Other _____ |
| Is there an EID register here? | [__] yes formal  [__] yes informal  [__] no | [__] yes formal  [__] yes informal  [__] no | [__] yes formal  [__] yes informal  [__] no | [__] yes formal  [__] yes informal  [__] no | [__] yes formal  [__] yes informal  [__] no |
| Total Infants Tested | Estimate y or n | Estimate y or n | Estimate y or n | Estimate y or n | Estimate y or n |
| How are samples transported? |  |  |  |  |  |
| Where are samples tested? |  |  |  |  |  |
| How long does it take to get your results back? |  |  |  |  |  |
| Have you not received any results? | [__] no  [__] yes  If yes, how many? | [__] no  [__] yes  If yes, how many? | [__] no  [__] yes  If yes, how many? | [__] no  [__] yes  If yes, how many? | [__] no  [__] yes  If yes, how many? |
| Total Infants received results | Estimate y or n | Estimate y or n | Estimate y or n | Estimate y or n | Estimate y or n |
| Total infants tested positive | Estimate y or n | Estimate y or n | Estimate y or n | Estimate y or n | Estimate y or n |
| Total positives received results | Estimate y or n | Estimate y or n | Estimate y or n | Estimate y or n | Estimate y or n |
| What happens when infants receive a positive result? |  |  |  |  |  |
| Biggest Challenge with EID |  |  |  |  |  |
| Other Comments |  |  |  |  |  |

**** LOOK NEXT PAGE

**Other PMTCT questions:**

___ PMTCT moms followed in 08

___ PMTCT moms followed thus far in 09

Have there been stock-outs of rapid tests for mothers on ANC?

[__] no

[__] yes

If yes for how long?

Is there a mechanism for enrolling mom’s testing positive at ANC at the ART center? *If yes, what?*

Is there a mechanism to follow up infants born from PMTCT mothers? *If yes, what?*

What other partners are active here? *Please list*

| **EID INTEGRATION INTO EPI OUTREACH and CDPs** | |
| --- | --- |
| 1. Have you integrated EID into CDPs | [__] Yes  [__] No  [__] Other ___________________________________ |
| 1. When did you start integration? |  |
| 1. Have you integrated EID into your regular outreach? | [__] Yes  [__] No  [__] Other ___________________________________ |
| 1. How many infants have you tested in outreach? |  |
| 1. How many of those infants received CTX? |  |
| 1. How many of those infants received their results? |  |
| 1. How were results returned? |  |
| 1. How many results were positive? |  |
| 1. How many have been referred to ART? |  |
| 1. Any other challenges? |  |
| 1. Any recommendations? |  |

**If this site does not provide pediatric ART services, HIV positive infants are referred where? _______________________**

If Site Provides Pediatric ART Services:

| **Adult and Pediatric Care and Treatment** | |
| --- | --- |
| 1. How many adults (15+) are on ART here? | **Q3** |
| 1. How many children (0-14yrs) are on ART here? | **Q3** |
| 1. What is the average age of ART initiation in children here? |  |
| 1. ***Roughly*** how many of the children followed are <5yr old | ______ in care  ______ on ART |
| 1. Which EID sites refer HIV positive infants to your care and treatment site? | (*list all)* |
| 1. **How many infants were enrolled when they were <18m? |  |
| 1. **How many infants were initiated on ART when they were <18m (from 2007 to date)? |  |
| 1. *Of those, how many are alive & active on ART from 2007 to date? | **Totals should match positive grid** |
| 1. **When** and **how** were you informed and trained of the **immediate treatment policy** for infants <12m? |  |
| 1. What challenges do you face with regards to infant treatment? |  |
| 1. What suggestions do you have to strengthen infant treatment? |  |
| **Clinical Care and Clinical Organization** | |
| 1. Do you have a system to monitor the schedule of appointments? | [__] Yes [__] No  *If yes, note the system____________________* |
| 1. Is there a mechanism to trace patients if they default once enrolled an ART center? | [__] Yes [__] No  *If yes, what _____________________________* |
| 1. Is the same support offered for people in pre-ART as for those on ART, for adults & for peds | [__] Yes [__] No  *If no, explain___________________________* |
| 1. Is pediatric loss to follow up a significant problem? | [__] Yes [__] No  ***If yes, why _____________________________*** |
| 1. What do you think is the most important advice for a successful EID scale-up |  |
| **ART Pharmacy** | |
| Have you had stock-outs of any EID consumables? | If yes, for how long  [__] ped ARVs ____________  [__] adult ARVs ____________  [__] CTX ____________  [__] Other OI meds ____________  [__] Other ____________  [__] Other ____________ |
